# Supplementary material for: Topological Distribution of Wound Stiffness Modulates Wound-Induced Hair Follicle Neogenesis
Source: Pharmaceutics. 2022 Sep 12;14(9):1926. doi: 10.3390/pharmaceutics14091926 (PMC9504897; doi:10.3390/pharmaceutics14091926)
Supplement: Supplementary file 1 [file pharmaceutics-14-01926-s001.zip › pharmaceutics-1875582-supplementary.pdf]

pharmaceutics

MDPI

Supplementary Materials: Topological Distribution of Wound Stiffness Modulates Wound-Induced Hair Follicle Neogenesis

Hans I-Chen Harn, Po-Yuan Chiu, Chein-Hong Lin, Hung-Yang Chen, Yung-Chih Lai, Fu-Shiuan Yang, Chia-Ching Wu, Ming-Jer Tang, Cheng-Ming Chuong and Michael W. Hughes

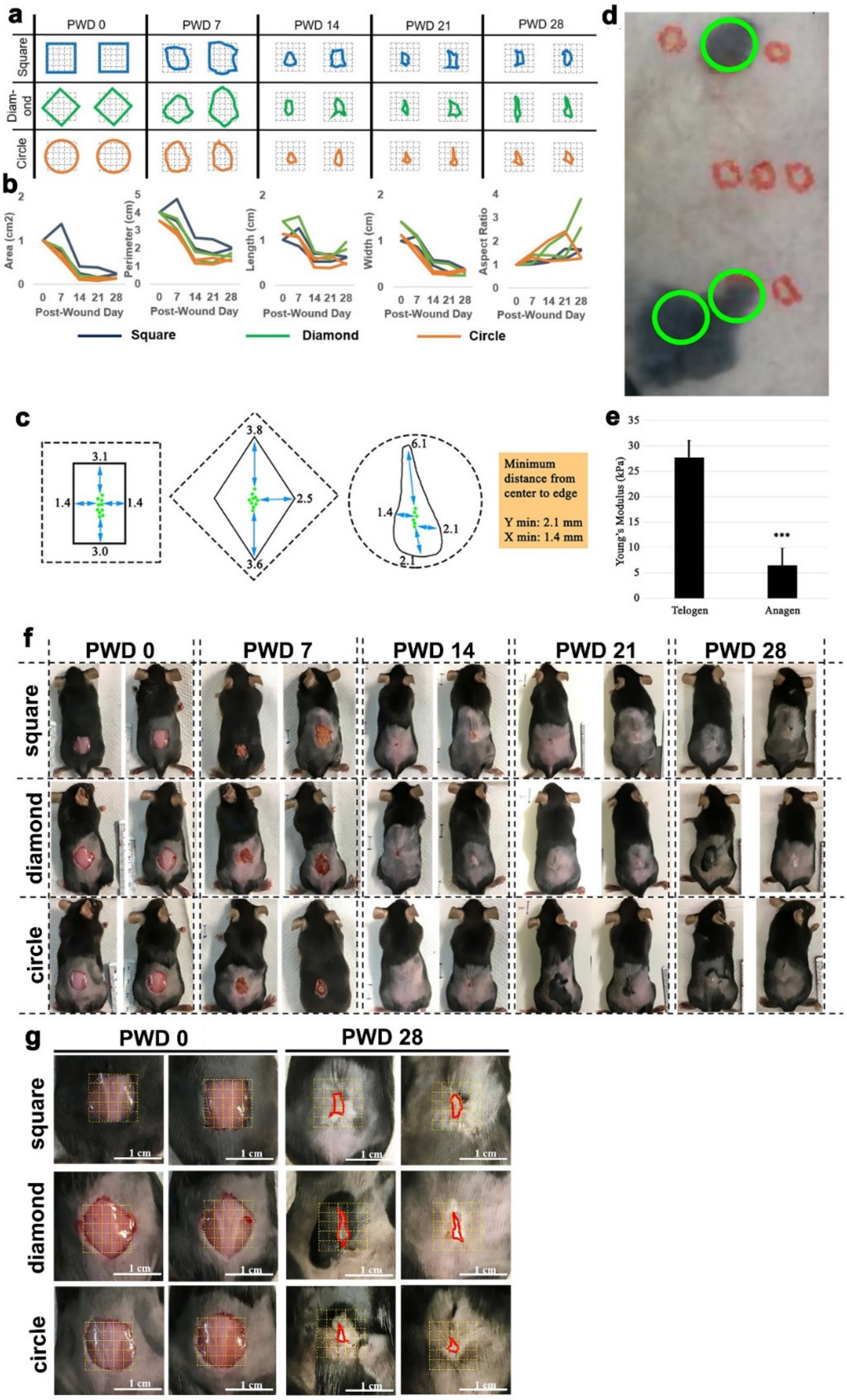

Figure S1. Wound geometry and hair cycle epidermal stiffness suggest a role for mechanobiology during wound induced hair follicle neogenesis. (**a**) Summary of wound shape change for square, diamond and circle wounds during WIHN. (colored lines; wound size and shape, gray 1 × 1 cm grid overlay; visualization aid for wound shape change) (**b**) Quantified parameters of area, perimeter, length, width and aspect ratio of square, diamond, and circle wounds during WIHN. (**c**) Initial (dotted line) and final shape (solid line) analysis for square, diamond, and circle wounds. (**d**) AFM was used to measure skin stiffness of anagen and telogen skin. (green circle; anagen, red circle; telogen) (**e**) Quantification of average epidermis stiffness during anagen or telogen. All measurements were taken using a cantilever with a 25 um diameter bead tip with an indentation force of 5 nN. Data are represented as mean ± SEM. ($n = 5$, *** $p < 0.001$) (**f**) Secondary intention wound healing time-course of 3-week-old C57Bl/6 mice for full-thickness 1 cm^2 square, diamond, or circle wounds. Photos were taken every 7 days from PWD 0 until 28. (**g**) Magnified photos PWD 0 and PWD 28 wounds. (scale bars; 1 cm, red lines; wound size and shape, 1 × 1 cm yellow grid overlay; visual aid for wound shape change).

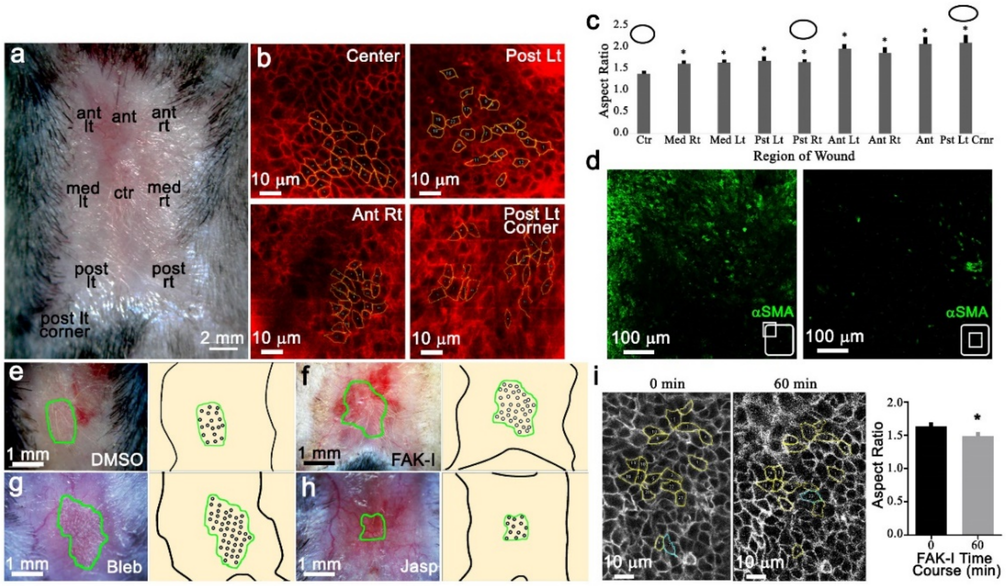

Figure S2. Epithelial cell shape, SMA patterning, and regenerative area during wound induced hair follicle neogenesis. (**a**) Wound positions used for cell shape analysis at PWD 21. (scale bar; 2 mm) (**b**) Representative confocal images of epidermal cell locations of live ROSAmT/mG mice at PWD 21. (scale bar; 10 μm, yellow outlines; epithelial cell membrane used for aspect ratio analysis by ImageJ) (**c**) Quantification of epithelial cell aspect ratio of 50 cells in 200 × 200 μm^2 area of the wound. (scale bar; 1 mm, black circles; cell shape changes with respect to aspect ratio, * $p < 0.05$) (**d**) Confocal image of compressed z-stack layers for α-SMA immunostaining in epidermis from PWD 14 wounds. (scale bar; 100 μm) (**e**-**h**) 1 cm^2 square wounds treated with (**e**) DMSO, (**f**) FAK-I, (**g**) Blebbistatin, or (**h**) Jasplakinolide during WIHN and respective summary diagrams at PWD 28. (green lines; regenerative area, black lines; wound edge, black dots; de novo follicles, $n = 4$, scale bar 1 mm). (**i**) 4D confocal imaging of PWD 14 ROSAmT/mG mice treated topically with 1 μM FAK-I and imaged in real time. Quantification of the aspect ratio of 50 cells in a 200 × 200 μm^2 area of the wound. (scale bar; 10 μm, data are represented as mean ± SD. ($n = 4$), *, $p < 0.05$).

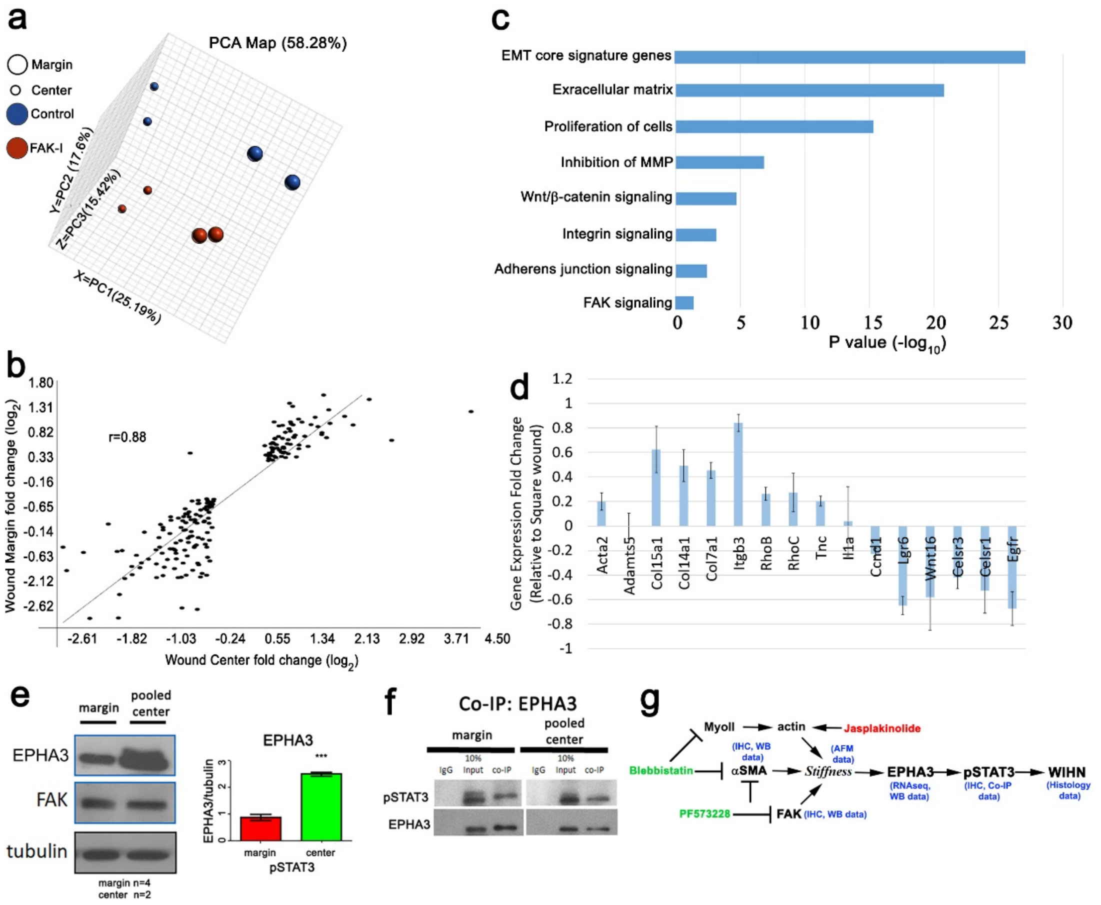

Figure S3. RNA-seq and quantitative PCR exhibit differential gene expression in topical FAK inhibitor treated wounds versus control. (**a**) Principal Component Analysis (PCA) identifies 4 sample groups according to data variance. (**b**) Linear regression analysis of; control wound center vs FAK-I treated wound center, and control wound margin vs FAK-I treated wound margin. (**c**) Differential expression analysis between wound center and margin in control PWD 14 wound epidermis. (**d**) qPCR analysis of 1 μM FAK-I daily treatment from PWD 10 to PWD 14 shows downregulated cytoskeleton, ECM, integrin signaling related genes, and upregulated hair follicular genesis and cell proliferation related genes in the wound epidermis compared to control samples. (**e**) Western blot protein expression patterns of EPHA3 and FAK from the wound margin versus center. (**f**) Immunoprecipitation blot of EPHA3 and pSTAT3. (**g**) Summary diagram of topical treatment experiments that enhanced (green text) or inhibited (red text) WIHN. (n = 3, SEM).
